# Sample size considerations for the external validation of a multivariable prognostic model: a resampling study

**DOI:** 10.1002/sim.6787

**Published:** 2015-11-09

**Authors:** Gary S. Collins, Emmanuel O. Ogundimu, Douglas G. Altman

**Affiliations:** ^1^ Centre for Statistics in Medicine, Nuffield Department of Orthopaedics, Rheumatology and Musculoskeletal Sciences, Botnar Research CentreUniversity of OxfordWindmill RoadOxfordOX3 7LDU.K.

**Keywords:** prognostic model, sample size, external validation

## Abstract

After developing a prognostic model, it is essential to evaluate the performance of the model in samples independent from those used to develop the model, which is often referred to as external validation. However, despite its importance, very little is known about the sample size requirements for conducting an external validation. Using a large real data set and resampling methods, we investigate the impact of sample size on the performance of six published prognostic models. Focussing on unbiased and precise estimation of performance measures (e.g. the *c*‐index, D statistic and calibration), we provide guidance on sample size for investigators designing an external validation study. Our study suggests that externally validating a prognostic model requires a minimum of 100 events and ideally 200 (or more) events. © 2015 The Authors. Statistics in Medicine Published by John Wiley & Sons Ltd.

## 1 Introduction

Prognostic models are developed to estimate an individual's probability of developing a disease or outcome in the future. A vital step toward accepting a model is to evaluate its performance on similar individuals separate from those used in its development, which is often referred to as external validation or transportability 1, 2. However, despite the widespread development of prognostic models in many areas of medicine 3, 4, 5, very few been externally validated 6, 7, 8.

To externally validate a model is to evaluate its predictive performance (calibration and discrimination) using a separate data set from that used to develop the model 9. It is not repeating the entire modelling process on new data, refitting the model to new ‘validation’ data, or fitting the linear predictor (prognostic index) from the original model as a single predictor to new data 9. It is also not necessarily comparing the *similarity* in performance to that obtained during the development of the prognostic model. Whilst in some instances a difference in the performance can be suggestive of deficiencies in the development study, the performance in the new data may still be sufficiently good enough for the model to be potentially useful.

The case‐mix (i.e., the distribution of predictors included in the model) will influence the performance of the model 10. It is generally unlikely that the external validation data set will have an identical case‐mix to the data used for development. Indeed, it is preferable to use a slightly different case‐mix in external validation to judge model transportability. Successful external validation studies in diverse settings (with different case‐mix) indicate that it is more likely that the model will be generalizable to plausibly related, but untested settings 11.

Despite the clear importance of external validation, the design requirements for studies that attempt to evaluate the performance of multivariable prognostic models in new data have been little explored 7, 12, 13. Published studies evaluating prognostic models are often conducted using sample sizes that are clearly inadequate for this purpose, leading to exaggerated and misleading performance of the prognostic model 7. Finding such examples is not difficult 14, 15, 16. For example, a modified Thoracoscore, to predict in‐hospital mortality after general thoracic surgery, was evaluated using 155 patients, but included only eight events (deaths). A high *c*‐index value was reported, 0.95 (95% confidence interval 0.91 to 0.99) 16. In the most extreme case, a data set with only one outcome event was used to evaluate a prognostic model 14. In this particular study, an absurd value of the *c*‐index was reported, 1.00 (95% confidence interval 1.00 to 1.00)[*sic*]. Concluding predictive accuracy, and thus that the model is fit for purpose, on such limited data is nothing but misleading.

The only guidance for sample size considerations that we are aware of is based on a hypothesis testing framework (i.e. to detect pre‐specified changes in the c‐statistic) and recommends that models developed using logistic regression are evaluated with a minimum of 100 events 12. However, a recent systematic review evaluating the methodological conduct of external validation studies found that just under half of the studies evaluated models on fewer than 100 events 7.

It is therefore important to provide researchers with appropriate guidance on sample size considerations when evaluating the performance of prognostic models in an external validation study. When validating a prognostic model, investigators should clearly explain how they determined their study size, so that their findings can be placed in context 17, 18. Our view is that external validation primarily concerns the accurate (unbiased) estimation of performance measures (e.g., the *c*‐index). It does not necessarily include formal statistical hypothesis testing, although this may be useful in some situations. Therefore sample size considerations should be based on estimating performance measures that are sufficiently close to the *true* underlying population values (i.e., unbiased) along with measures of uncertainty that are sufficiently narrow (i.e., precise estimates) so that meaningful conclusions on the model's predictive accuracy in the target population can be drawn 9, 19.

The aim of this article is to examine sample size considerations for studies that attempt to externally validate prognostic models and to illustrate that many events are required to provide reasonable estimates of model performance. Our study uses published prognostic models (QRISK2 20, QDScore 21 and the Cox Framingham risk score 22) to illustrate sample size considerations using a resampling design from a large data set (>2 million) of general practice patients in the UK.

The structure of the paper is as follows. Section 2 describes the clinical data set and the prognostic models. Section 3 describes the design of the study, the assessment of predictive performance and the methods used to evaluate the resampling results. Section 4 presents the results from the resampling study, which are then discussed in Section 5.

## 2 Data Set and prognostic models

### 2.1 Study data: the health improvement network

The Health Improvement Network (THIN) is a large database of anonymized primary care records collected at general practice surgeries around the UK. The THIN database currently contains medical records on approximately 4% of the UK population. Clinical information from over 2 million individuals (from 364 general practices) registered between June 1994 and June 2008 form the data set. The data have previously been used in the external validation of a number of prognostic models (including those considered in this study) 23, 24, 25, 26, 27, 28, 29, 30. There are missing data for various predictors needed to use the prognostic models. For simplicity, we have used one of the imputed data sets from the published external validation studies, where details on the imputation strategy can be found 23, 24.

### 2.2 Prognostic models

At the core of the study are six sex‐specific published models for predicting the 10‐year risk of developing cardiovascular disease (CVD) (QRISK2 20, and Cox Framingham 22) and the 10‐year risk of developing type 2 diabetes (QDScore 21). All six prognostic models are all predicting time‐to‐event outcomes using Cox regression. None of these models were developed using THIN, but THIN has previously been used to evaluate their performance in validation studies 23, 24.

QRISK2 was developed using 1.5 million general practice patients aged between 35 and 74 years (10.9 million person years of observation) contributing 96 709 cardiovascular events from the QRESEARCH database 20. Separate models are available for women (41 042 CVD events) and men (55 667 CVD events), containing 13 predictors, 8 interactions and fractional polynomial terms for age and body mass index (www.qrisk.org).

Cox Framingham was developed using 8491 Framingham study participants aged 30 to 74 years contributing 1274 cardiovascular events 22. Separate models are available for women (456 CVD events) and men (718 CVD events), each containing 7 predictors.

QDScore was developed on 2.5 million general practice patients aged between 25 and 79 years (16.4 million person years of observation) contributing 72 986 incident diagnoses of type 2 diabetes from the QRESEARCH database 21. Separate models are available for women and men, each containing 12 predictors, 3 interactions and fractional polynomial terms for age and body mass index (www.qdscore.org).

## 3 Methods

### 3.1 Resampling strategy

A resampling strategy was applied to examine the influence of sample size (more specifically, the number of events) on the bias and precision in evaluating the performance of published prognostic models.

Samples were randomly drawn (with replacement) from the THIN data set so that the number of events in each sample was fixed at 5, 10, 25, 50, 75, 100, 150, 200, 300, 400, 500 or 1000 by stratified sampling according to the outcome ensuring that the proportion of events in each sample was the same as the overall proportion of events in the THIN data set (Table 1). The sample sizes for each prognostic model at each value of number of events can be found in the Supporting Information. For each scenario (i.e., for each sample size), 10 000 samples (denoted *B*) were randomly drawn and performance measures were calculated for each sample.

**Table 1:** ‘True’ values based on the entire THIN validation cohort.

		Number of individuals	Number of events (%)	Performance measure					
				*c*‐index	*D* statistic	$R_{D}^{2}$	$\rho_{OXS}^{2}$	Brier score	Calibration slope
QRISK2 20, 51	Women	797,373	29,507 (3.64)	0.792	1.650	0.394	0.668	0.052	0.948
	Men	785,733	42,408 (5.40)	0.775	1.530	0.359	0.607	0.075	1.000
Cox Framingham 22	Women	797,373	29,507 (3.64)	0.756	1.435	0.330	0.553	0.055	0.919
	Men	785,733	42,408 (5.40)	0.759	1.452	0.335	0.554	0.084	1.001
QDScore 21, 23	Women	1,211,038	32,200 (2.66)	0.810	1.872	0.456	0.731	0.041	0.875
	Men	1,185,354	40,786 (3.44)	0.800	1.760	0.425	0.687	0.053	0.869

### 3.2 Performance measures

The performance of the prognostic models was quantified by assessing aspects of model discrimination (the *c*‐index 31 and *D* statistic 32), calibration 9, 33, and other performance measures ( $R_{D}^{2}$ 34, $R_{OXS}^{2}$ 35 and the Brier score for censored data 36).

Discrimination is the ability of a prognostic model to differentiate between people with different outcomes, such that those without the outcome (e.g., alive) have a lower predicted risk than those with the outcome (e.g., dead). For the survival models used within this study, which are time‐to‐event based, discrimination is evaluated using Harrell's *c*‐index, which is a generalization of the area under the receiver operating characteristic curve for binary outcomes (e.g., logistic regression) 31, 37. Harrell's *c*‐index can be interpreted as the probability that, for a randomly chosen pair of patients, the patient who actually experiences the event of interest earlier in time has a lower predicted value. The *c*‐index and its standard error were calculated using the rcorr.cens function in the rms library in R.

We also examined the *D* statistic, which can be interpreted as the separation between two survival curves (i.e., a difference in log HR) for two equal size prognostic groups derived from Cox regression 32. It is closely related to the standard deviation of the prognostic index (*PI* = *β*_1_ *x*_1_ + *β*_2_ *x*_2_ + ⋯ + *β*_*k*_ *x*_*k*_), which is a weighted sum of the variables (*x_i_*) in the model, where the weights are the regression coefficients (*β_i_*). *D* is calculated by ordering the values from the prognostic index, transforming them using expected standard normal order statistics, dividing the result by $\kappa =\sqrt{8/\pi }≃1.596$ and fitting this in a single term Cox regression. *D* and its standard error are given by the coefficient and standard error in the single term Cox regression model.

The calibration slope was calculated by estimating the regression coefficient in a Cox regression model with the prognostic index (the linear predictor) as the only covariate. If the slope is <1, discrimination is poorer in the validation data set (regression coefficients are on average smaller than the development data set), and conversely, it is better in the validation data set if the slope is >1(regression coefficients are on average larger than the development data set) 9, 33. We also examined the calibration of the models over the entire probability range at a single time point (at 10 years) using the val.surv function in the rms library in R, which implements the hare function from the polspline package for flexible adaptive hazard regression 38, 39. In summary, for each random sample, hazard regression using linear splines are used to relate the predicted probabilities from the models at 10 years to the observed event times (and censoring indicators) to estimate the actual event probability at 10 years as a function of the estimate event probability at 10 years. To investigate the influence of sample size on calibration, for each event size, plots of observed outcomes against predicted probabilities were drawn and overlaid for each of the 10 000 random samples.

We examined two *R* ^2^‐type measures 40, 41 (explained variation 32 and explained randomness 35) and the Brier score 42. Royston and Sauerbrei's $R_{D}^{2}$ is the proportion of the that is explained by the prognostic model 32, 34 and is given by

$$R_{D}^{2}=\frac{D^{2}/\kappa^{2}}{\sigma^{2}+D^{2}/\kappa^{2}}$$

where *D* is the value of the *D* statistic 32, σ^2^ = *π* ^2^/6 ≃ 1.645 and $\kappa =\sqrt{8/\pi ≃1.596}$. The measure of explained randomness, $\rho_{k}^{2}$ of O'Quigley *et al*. 35 is defined as

$$\rho_{OXS}^{2}=1-\exp\left\{-\frac{2}{k}\left(l_{\beta }-l_{0}\right)\right\}$$

where *k* is the number of outcome events, and *l_b_* and *l*_0_ are the log partial likelihoods for the prognostic model and the null model respectively. Standard errors of $\rho_{k}^{2}$ were calculated using the nonparametric bootstrap (200 bootstrap replications).

The Brier score for survival data is a measure of the average discrepancy between the true disease status (0 or 1) and the predicted probability of developing the disease 36, 43, defined as a function of time *t* > 0:

$$BS\left(t\right)=\frac{1}{n}\sum_{i=1}^{n}\left[\frac{\hat{S}{\left(t|X_{i}\right)}^{2}\cdot I\left(t_{i}\leq t,\;\delta_{i}=1\right)}{Ĝ\left(t_{i}\right)}+\frac{{\left(1-\hat{S}\left(t|X_{i}\right)\right)}^{2}\cdot I\left(t_{i}>t\right)}{Ĝ\left(t\right)}\right]$$

where *Ŝ*(· |*X*_*i*_) is the predicted probability of an event for individual *i*; *Ĝ is* the Kaplan–Meier estimate of the censoring distribution, which is based on the observations (*t*_*i*_, 1 − *δ*_*i*_),*δ*_*i*_ is the censoring indicator and *I* denotes the indicator function. 36, 43, 44. The Brier score is implemented in the function sbrier from the package ipred in R.

### 3.3 Evaluation

The objective of our study was to evaluate the impact of sample size (more precisely the number of events) on the accuracy, precision and variability of model performance. We examined the sample size requirements using the guidance by Burton *et al*. 45. We calculated the following quantities for each of the performance measures over the *B* simulations (defined in the preceding section):

- Percentage bias, which is the relative magnitude of the raw bias to the true value, defined as $\left(\hat{\theta }--\theta \right)/\theta$.
- Standardized bias, which is the relative magnitude of the raw bias to the standard error, defined as $\left(\hat{\theta }--\theta \right)/SE\left(\hat{\theta }\right)$. A standardized bias of −25 percent implies that the estimate lies one quarter of a standard error below the true value.
- Root mean square error, which incorporates both measures of bias and variability of the estimate, defined as $\sqrt{\frac{1}{B}\sum_{i=1}^{B}{\left({\hat{\theta }}_{i}-\theta \right)}^{2}}$.
- Estimated coverage rate of the 95% confidence interval for the *c*‐index, *D* statistic, $R_{D}^{2}$ and $\rho_{OXS}^{2}$, which indicate the proportion of times that a confidence interval contains the true value (*θ*). An acceptable coverage should not fall outside of approximately two standard errors of the nominal coverage probability $\left(p\right),\;SE\left(p\right)=\sqrt{p\left(1-p\right)/B}$ 46.
- Average width of the confidence interval, defined as $1/B\left\{\sum_{i=1}^{B}2Z_{1-\alpha /2}SE\left({\hat{\theta }}_{i}\right)\right\}$.

The *true* values (*θ*) of the performance measures were obtained using the entire THIN data set for each model (Table 1). $\hat{\theta }=\sum_{i=1}^{B}{\hat{\theta }}_{i}/B$, where *B* is the number of simulations performed and ${\hat{\theta }}_{i}$ is the performance measure of interest for each of the *i* = 1,…,*B* = 10 000 simulations. The empirical standard error, $SE\left(\hat{\theta }\right)$, is the square root of the variance of over all *B*‐simulated $\hat{\theta }$ values. If, for the *D* statistic and $R_{D}^{2}$, the model‐based standard error is valid, then its mean over the 10 000 simulations should be close to the empirical standard error $SE\left(\hat{\theta }\right)$.

## 4 Result

**Figure 1:**
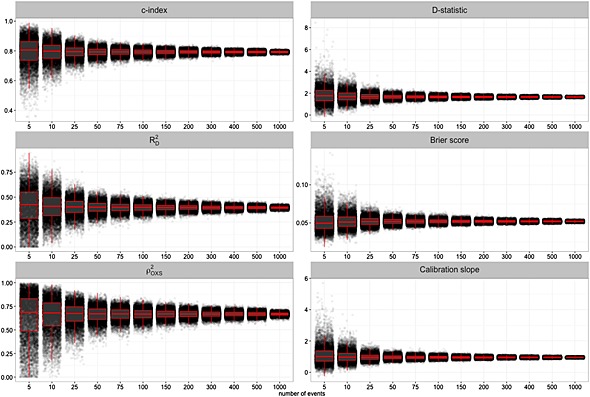
Empirical performance of QRISK2 (women), measured using the *c*‐index, *D* statistic, $R_{D}^{2}$, $\rho_{OXS}^{2}$, Brier score and calibration slope.

Figure 1 presents the empirical values, with boxplots overlaid, for the *c*‐index, *D* statistic, $R_{D}^{2}$, $\rho_{OXS}^{2}$ Brier score and calibration slope for QRISK2 (women), describing pure sampling variation. As expected, considerable variation in the sample values for each of the six performance measures are observed when the number of events is small. Thus, inaccurate estimation of the true performance is more likely in studies with low numbers of events.

**Figure 2:**
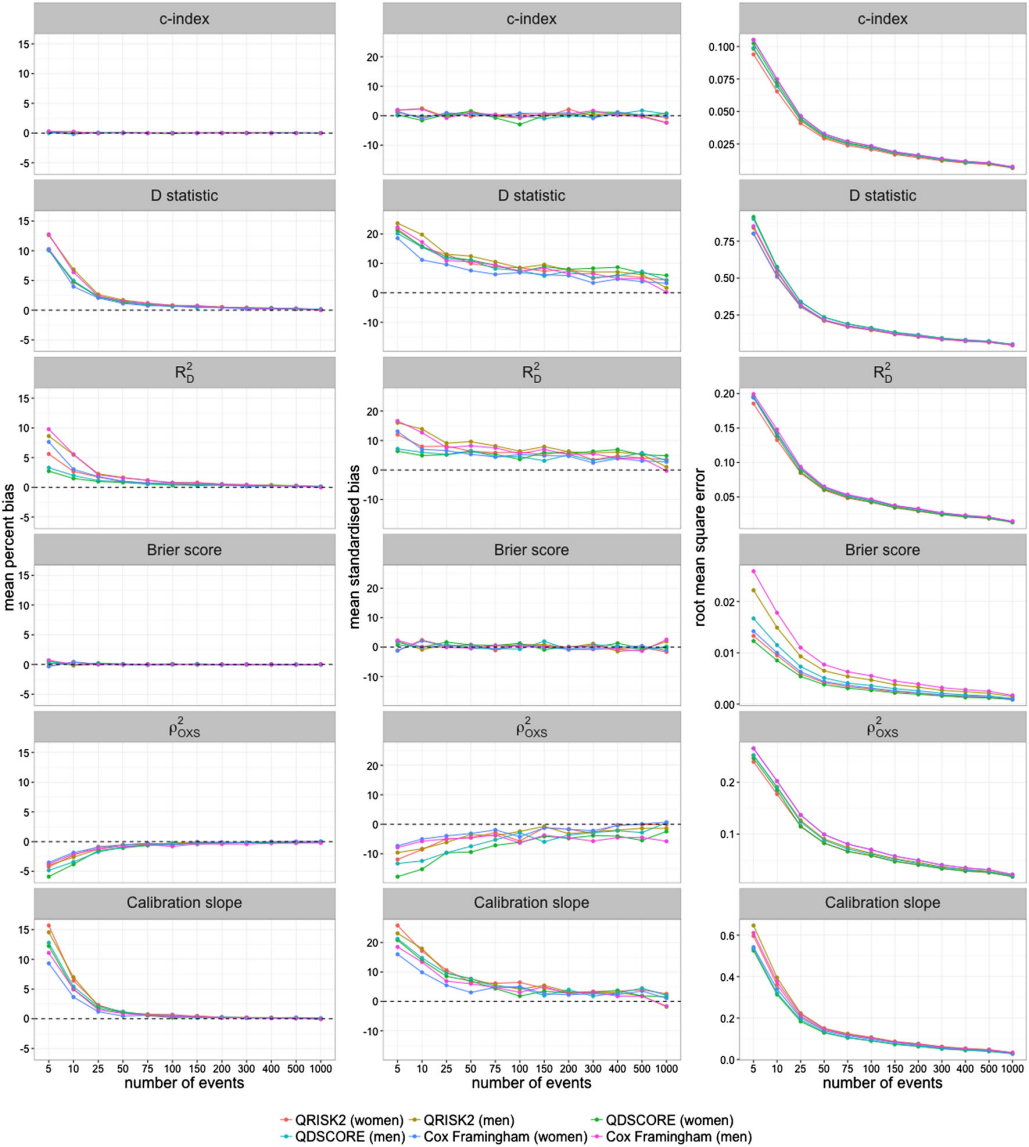
Mean percent, standardized bias and RMSE of the *c*‐index, *D* statistic, $R_{D}^{2}$, $\rho_{OXS}^{2}$, Brier score and calibration slope.

The mean percent bias, standardized bias and RMSE of the performance measures are displayed graphically in Figure 2. For all of the models, the mean percent bias of both the *c*‐index and Brier score are within 0.1% when the number of events reaches 50. At 50 events, the average bias of the *D* statistic, $R_{D}^{2}$ and calibration slope is within 2% of the true value. The mean standardized bias for all of the models and performance measures drops below 10% once the number of events increases to 75–100.

Because of the skewness in bias at small values of number of events, the median percent bias and standardized bias of the performance measures are also presented (Supporting Information). For all of the performance measures, the median bias drops below 1% as the number of events reaches 100. Similarly, the median standardized bias drops below 10% for all of the performance measures and models when the number of events approaches 100.

As expected, the RMSE decreases as the number of events increases for all six performance measures (Figure 2). The same pattern is observed for all six prognostic models.

**Figure 3:**
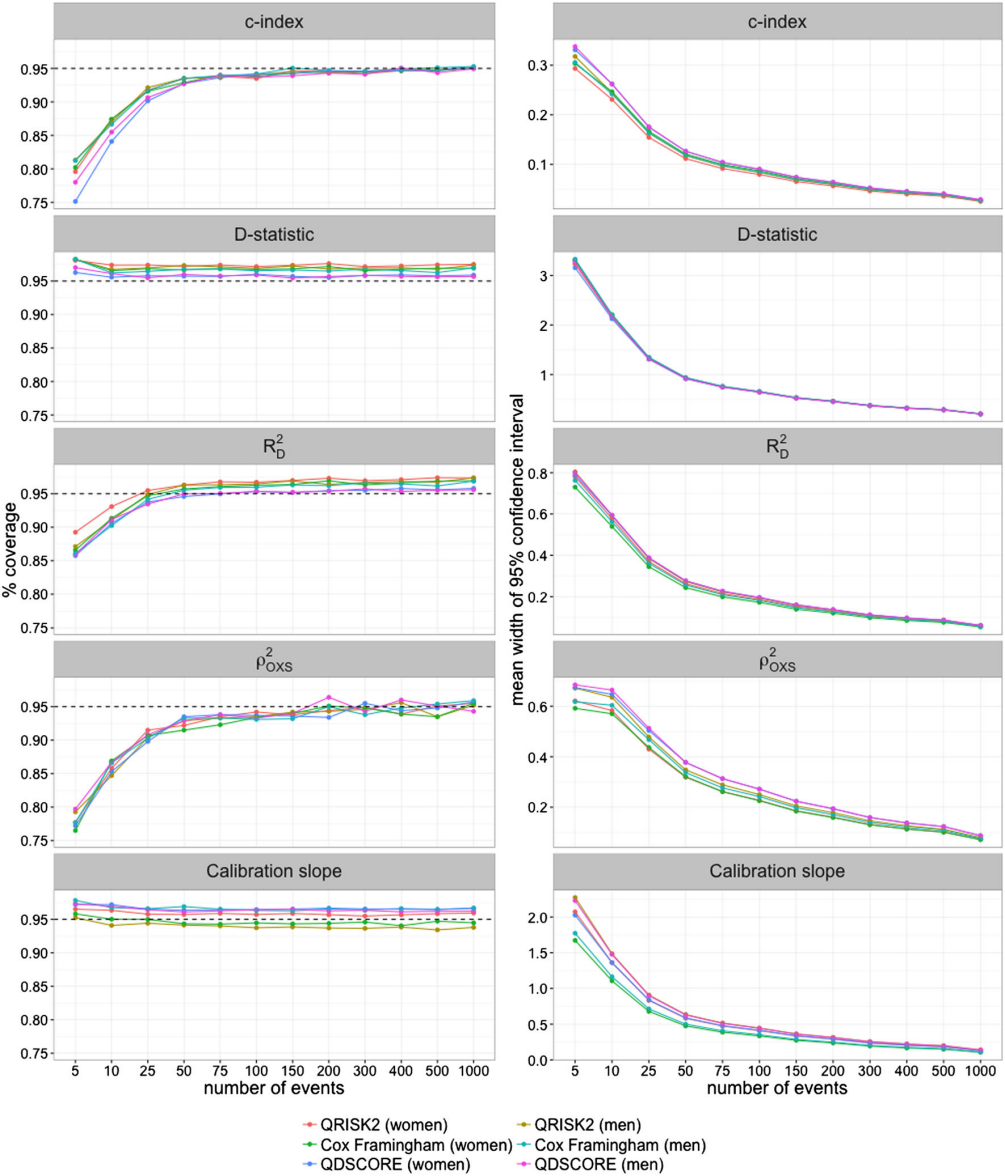
Coverage rates and 95% confidence interval widths for the *c*‐index, *D* statistic, $R_{D}^{2}$, $\rho_{OXS}^{2}$ and calibration slope. [Bootstrap standard errors for $\rho_{OXS}^{2}$ based on 1000 simulations and 200 bootstrap replications].

**Figure 4:**
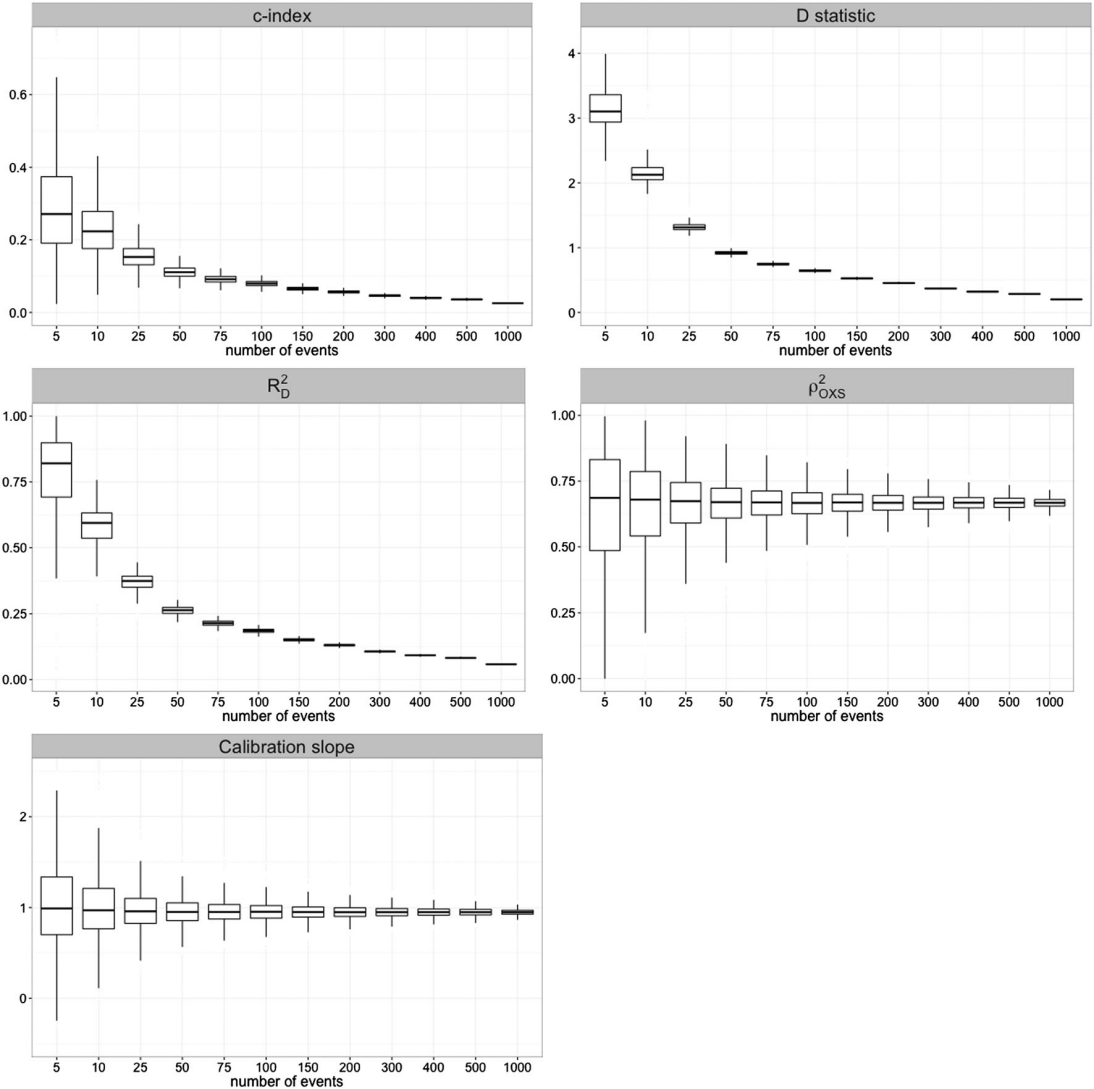
Width of the 95% confidence interval of the *c*‐index, *D* statistic $R_{D}^{2}$, $\rho_{OXS}^{2}$ and calibration slope (QRISK2 women). [Bootstrap standard errors for $\rho_{OXS}^{2}$ based on 1000 simulations and 200 bootstrap replications].

Coverage of the confidence intervals for the *c*‐index, *D* statistic and $R_{D}^{2}$ are displayed in Figure 3. Acceptable coverage of the *c*‐index at the nominal level of 95 percent is achieved as the number of events approaches and exceeds 200. However, the *D* statistic confidence interval exhibits over‐coverage regardless of sample size. There is under‐coverage of $R_{D}^{2}$ at less than 25 events and over‐coverage as the number of events increases (for four of the six prognostic models examined). The mean widths of the 95% confidence intervals for all of the models are displayed in Figure 3. A steep decrease is observed in the mean width for all models as the number of events approaches 50–100. Within this range, the decrease in mean width becomes smaller with more events. A similar pattern is observed in the width variability, as shown in Figure 4 for QRISK2 (women).

**Figure 5:**
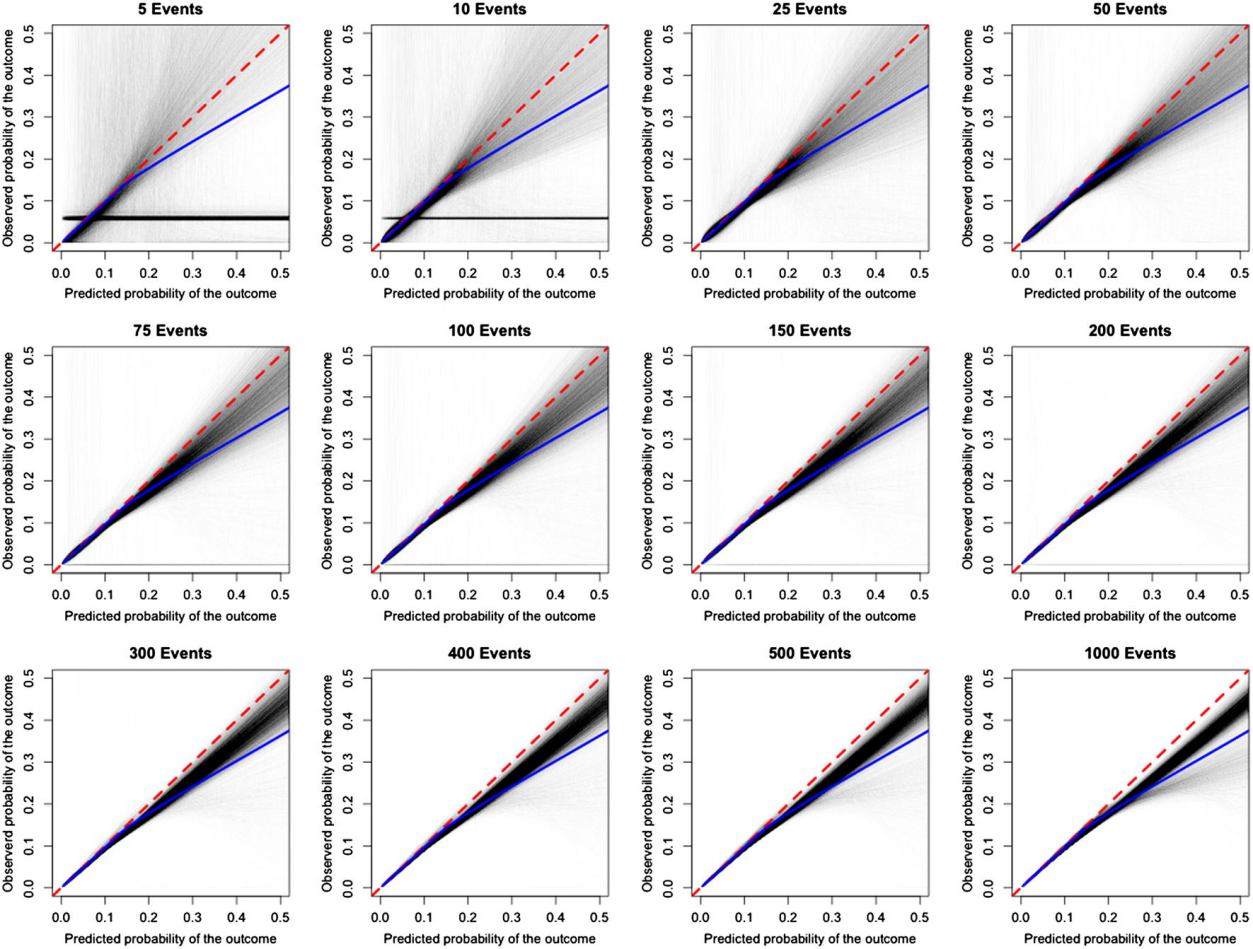
Calibration plots for QRISK2 (women). The red dashed line denoted perfect prediction. The blue line is the model calibration using the entire data set.

The effect of sample size on the performance of the hazard regression assessment of calibration of QRISK2 (women) is described in Figure 5. For each panel (i.e., each event size), 10 000 calibration lines have been plotted and a diagonal (dashed) line going through the origin with slope 1 has been superimposed, which depicts perfect calibration. Furthermore, we have overlaid a calibration line using the entire THIN data set to judge convergence of increasing event size. For data sets with 10 or fewer numbers of events, the ability to assess calibration was poor. For predicted probabilities greater than 0.2, there was modest to substantial variation between the fitted calibration curves, which decreased as the number of events increased. The calibration line (blue line) using the entire THIN data set shows overestimation towards the upper tail of the distribution, whilst some overestimation is captured, from event sizes in excess of 100, the true magnitude of overestimation in using QRISK2 (women) in the THIN data set is not fully captured even when the number of events reach 1000. Calibration plots for two of the five prediction models (QRISK2 men and Cox Framingham women) show similar patterns, whilst for the remaining three models accurate assessment of calibration is achieved when the number of events reach 100 (data not shown).

**Figure 6:**
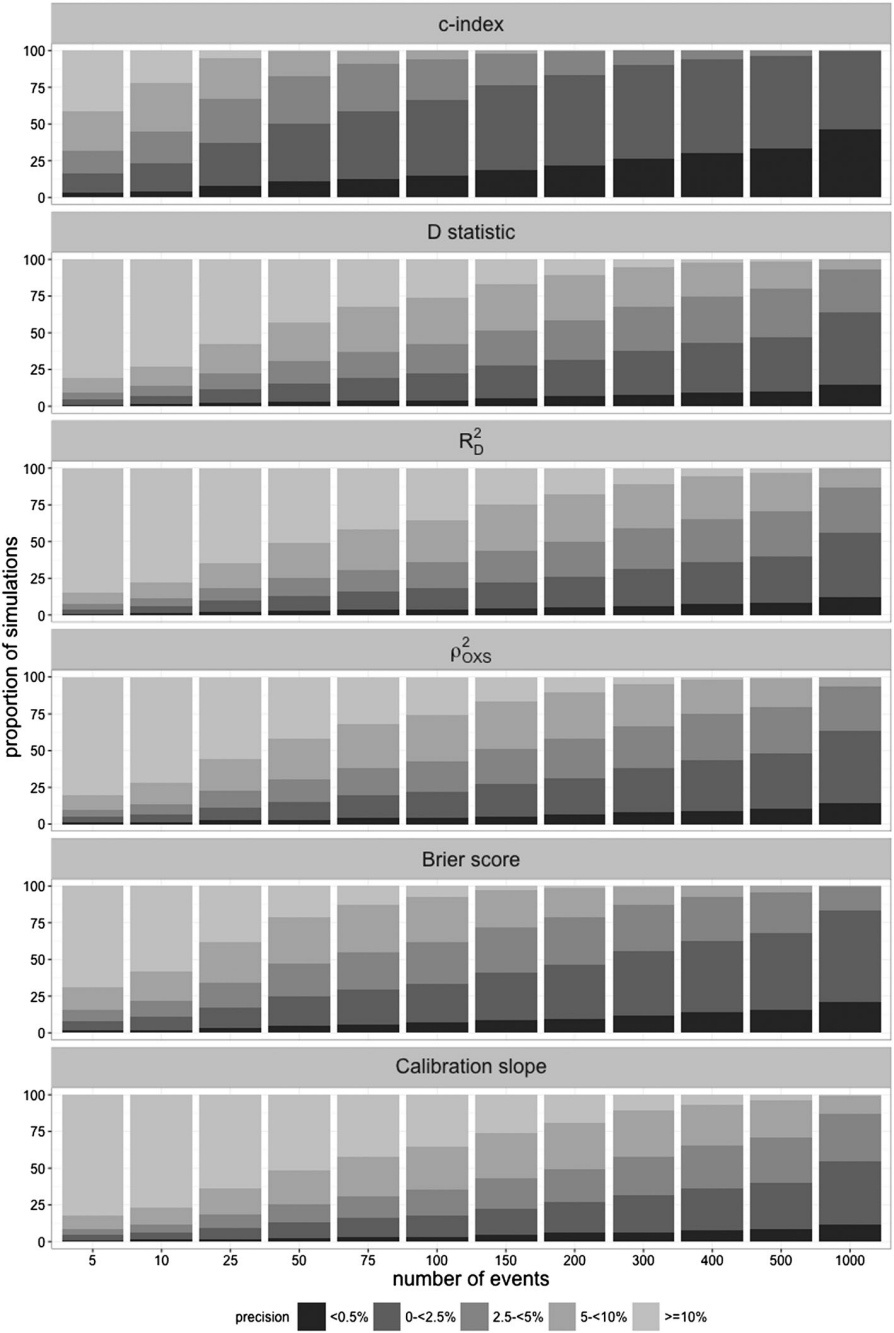
Proportion of estimates within 0.5, 2.5, 5, 1 and 0% of the true value for QRISK2 (women).

Figure 6 displays the proportion of simulations in which the performance estimates are within 0.5, 2.5, 5 and 10% of the true performance measure as the number of events increases. Fewer events are required to obtain precise estimates of the *c*‐index than of the other performance measures. For example, at 100 events, over 80% of simulations yield estimates of the *c*‐index within 5% of the true value and over 60% of simulations yield values within 2.5% of the true value. Considerably more events are required for the *D* statistic, $R_{D}^{2}$, Brier score and calibration slope.

### 4.1 Additional analyses

As observed in Figure 3, coverage of the *D* statistic is larger than the nominal 95% level regardless of the number of events. Similarly, $R_{D}^{2}$ coverage tends to be larger than the nominal 95% level as the number of events increases. Therefore, we carried out further analyses to investigate the model‐based standard error and the nonparametric bootstrap standard error of the *D* statistic and $R_{D}^{2}$ 47. The results are shown in Table 2.

**Table 2:** Standard errors (QRISK2 men) of the *D* statistic and $R_{D}^{2}$ based on 1000 simulations and 500 bootstrap replications.

Number of events (non‐events)	*D* statistic			$R_{D}^{2}$		
	Model‐based standard error	Empirical standard error	Bootstrap standard error	Model‐based standard error	Empirical standard error	Bootstrap standard error
10 (175)	0.5587	0.5424	0.5905	0.1508	0.1441	0.1334
25 (438)	0.3384	0.3046	0.3150	0.0983	0.0890	0.0874
50 (876)	0.2362	0.2163	0.2159	0.0696	0.0635	0.0623
75 (1315)	0.1914	0.1708	0.1730	0.0567	0.0506	0.0505
100 (1753)	0.1651	0.1481	0.1491	0.0492	0.0440	0.0440
200 (3506)	0.1162	0.1077	0.1046	0.0347	0.0322	0.0311
300 (5258)	0.0945	0.0853	0.0850	0.0283	0.0256	0.0254
400 (7011)	0.0819	0.0722	0.0735	0.0246	0.0216	0.0220
500 (8764)	0.0731	0.0656	0.0658	0.0219	0.0197	0.0197
1000 (17528)	0.0516	0.0463	0.0464	0.0155	0.0139	0.0139

The results from the additional simulations indicate that the model‐based standard error is overestimated. There is good agreement between the empirical and bootstrap standard errors, with coverage using the bootstrap standard errors close to the nominal 95 percent (Table 3).

**Table 3:** Coverage (QRISK2 men) based on model‐based and bootstrap standard errors for the *D* statistic and $R_{D}^{2}$ (1000 simulations; 500 bootstrap replications).

Number of events (non‐events)	*D* statistic		$R_{D}^{2}$	
	Model‐based standard error	Bootstrap standard error	Model‐based standard error	Bootstrap standard error
10 (175)	0.968	0.952	0.906	0.884
25 (438)	0.970	0.953	0.951	0.932
50 (876)	0.967	0.945	0.957	0.933
75 (1315)	0.974	0.950	0.965	0.942
100 (1753)	0.959	0.943	0.953	0.937
200 (3506)	0.966	0.941	0.965	0.935
300 (5258)	0.970	0.950	0.970	0.946
400 (7011)	0.976	0.952	0.975	0.953
500 (8764)	0.971	0.950	0.970	0.947
1000 (17528)	0.965	0.949	0.965	0.948

## 5 Discussion

External validation studies are a vital step in introducing a prognostic model, as they evaluate the performance and transportability of the model using data that were not involved in its development 2, 48. The performance of a prognostic model is typically worse when evaluated on samples independent of the sample used to develop the model 49. Therefore, the more external validation studies that demonstrate satisfactory performance, the more likely the model will be useful in untested populations, and ultimately, the more likely it will be used in clinical practice. However, despite their clear importance, multiple (independent) external validation studies are rare. Many prognostic models are only subjected to a single external validation study and are abandoned if that study gives poor results. Other investigators then proceed in developing yet another new model, discarding previous efforts, and the cycle begins again 2. However, systematic reviews examining methodological conduct and reporting have shown that many external validation studies are fraught with deficiencies, including inadequate sample size 7, 49. The results from our study indicate that small external validation studies are unreliable, inaccurate and possibly biased. We should avoid basing the decision to discard or recommend a prognostic model on an external validation study with a small sample size.

An alternative approach that could be used to determine an appropriate sample size for an external validation study is to focus on the ability to detect a clinically relevant deterioration in model performance 12. Whilst this approach may seem appealing, it requires the investigator to pre‐specify a performance measure to base this decision on and to justify the amount of deterioration that will indicate a lack of validation. Neither of these conditions are necessarily straightforward, particularly when the case‐mix is different or the underlying population in the validation data set is different to that from which the model was originally developed 50. We take the view that a single external validation is generally insufficient to warrant widespread recommendation of a prognostic model. The case‐mix in a development sample does not necessarily reflect the case‐mix of the intended population for which the model is being developed, as studies developing a prognostic model are rarely prospective and typically use existing data collected for an entirely different purpose. A prognostic model should be evaluated on multiple validation samples with different case‐mixes from the sample used to develop the model, thereby allowing a more thorough investigation into the performance of the model, possibly using meta‐analysis methods.

A strength of our study is the use of large data sets, multiple prognostic models and evaluating seven performance measures (*c*‐index, D statistic, $R_{D}^{2}$, $\rho_{OXS}^{2}$, brier score, calibration slope and calibration plots). We also showed that the analytical standard error for the D statistic (and $R_{D}^{2}$) are too large, but could be rectified by calculating bootstrap standard errors.

Fundamental issues in the design of external validation studies have received little attention. Existing studies examining the sample size requirements of multivariable prognostic models have focused on models developed using logistic regression 12, 13. Adopting a hypothesis testing framework, Vergouwe and colleagues suggested that a minimum of 100 events and 100 non‐events are required for external validation of prediction models developed using logistic regression 12. Peek and colleagues examined the influence of sample size when comparing multiple prediction models, including examining the accuracy of performance measures, and concluded that a substantial sample size is required 13. Our study took the approach that the sample size of an external validation study should be guided by the premise of producing accurate and precise estimates of model performance that reasonably reflect the true underlying population estimate. Despite the differences taken in approach, our recommendations coincide. Our study focused on prognostic models predicting time‐to‐event outcomes, whilst we don't expect any discernable differences, further studies are required to evaluate models predicting binary events. We suggest that externally validating a prognostic model requires a minimum of 100 events, preferably 200 or more events.

## Supporting information

Supporting info item

Supporting info item
