# Supplementary figures and images for: Sample size considerations for the external validation of a multivariable prognostic model: a resampling study
Source: Stat Med. 2015 Nov 9;35(2):214–26. doi: 10.1002/sim.6787 (PMC4738418; doi:10.1002/sim.6787)

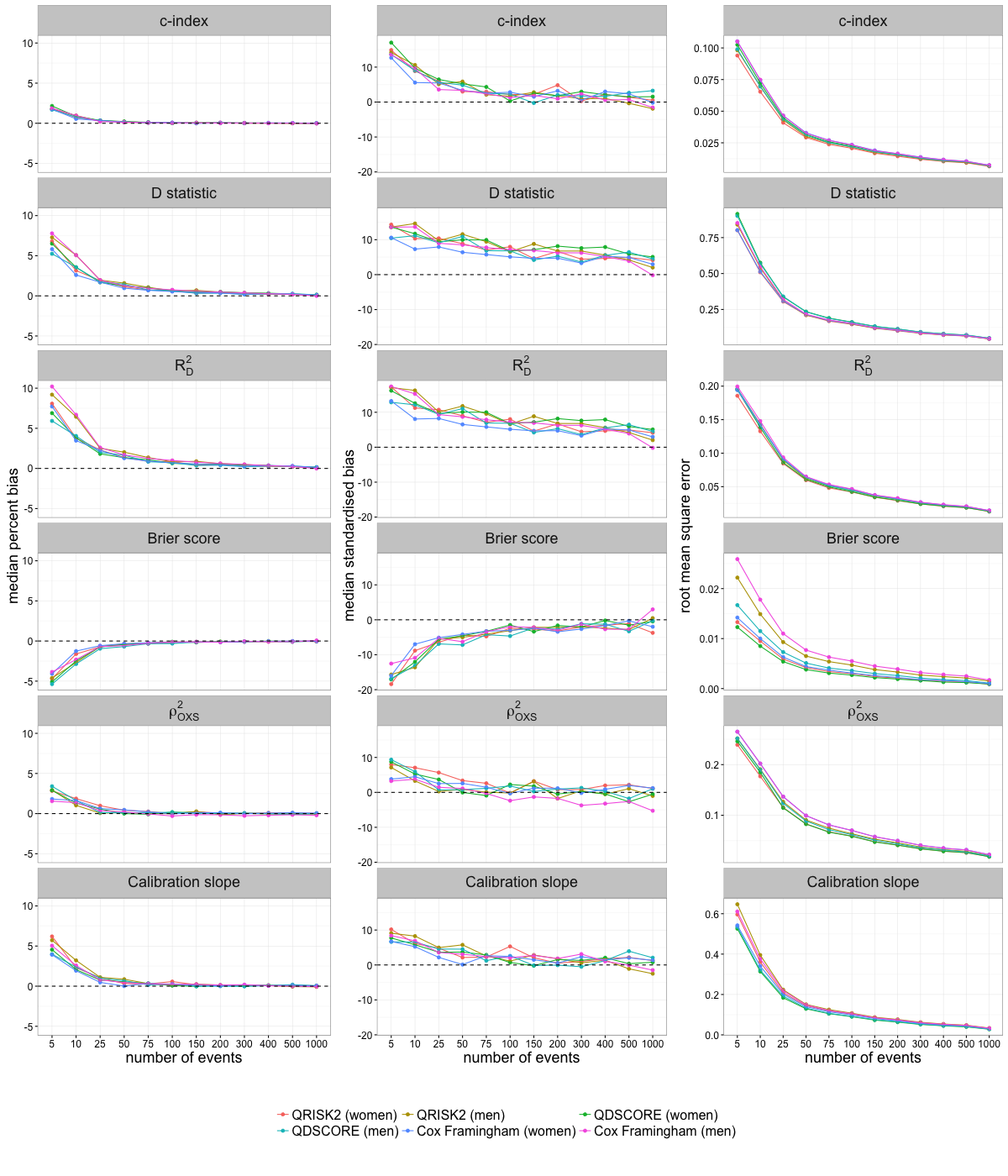

Supplement: Supplementary file 1 — Supporting info item [file SIM-35-214-s001.png]
