# Supplementary material for: Sample size considerations for the external validation of a multivariable prognostic model: a resampling study
Source: Stat Med. 2015 Nov 9;35(2):214–26. doi: 10.1002/sim.6787 (PMC4738418; doi:10.1002/sim.6787)
Supplement: Supplementary file 2 — Supporting info item [file SIM-35-214-s002.docx]

| **Outcome** | | **Number of outcome events** | | | | | | | | | | | |
| --- | --- | --- | --- | --- | --- | --- | --- | --- | --- | --- | --- | --- | --- |
|  |  | **5** | **10** | **25** | **50** | **75** | **100** | **150** | **200** | **300** | **400** | **500** | **1000** |
| **CVD** | **Men** | 88 | 175 | 438 | 876 | 1315 | 1753 | 2629 | 3506 | 5258 | 7011 | 8764 | 17528 |
|  | **Women** | 132 | 264 | 661 | 1322 | 1983 | 2644 | 3966 | 5288 | 7933 | 10577 | 13221 | 26442 |
| **Diabetes** | **Men** | 140 | 281 | 702 | 1403 | 2105 | 2806 | 4209 | 5613 | 8419 | 11225 | 14031 | 28063 |
|  | **Women** | 183 | 366 | 915 | 1830 | 2746 | 3661 | 5491 | 7322 | 10983 | 14644 | 18305 | 36610 |
